# Using Machine Learning Modeling to Explore New Immune-Related Prognostic Markers in Non-Small Cell Lung Cancer

**DOI:** 10.3389/fonc.2020.550002

**Published:** 2020-10-30

**Authors:** Jiasheng Xu, Han Nie, Jiarui He, Xinlu Wang, Kaili Liao, Luxia Tu, Zhenfang Xiong

**Affiliations:** ^1^ Department of Pathology, The First Affiliated Hospital of Nanchang University, Nanchang, China; ^2^ Department of Vascular Surgery, The Second Affiliated Hospital of Nanchang University, Nanchang, China; ^3^ Department of Clinical Laboratory, The Second Affiliated Hospital of Nanchang University, Nanchang, China

**Keywords:** machine learning, model building, immune-related prognostic markers, immunohistochemistry, non-small cell lung cancer

## Abstract

**Objective:**

To find new immune-related prognostic markers for non-small cell lung cancer (NSCLC).

**Methods:**

We found GSE14814 is related to NSCLC in GEO database. The non-small cell lung cancer observation (NSCLC-OBS) group was evaluated for immunity and divided into high and low groups for differential gene screening according to the score of immune evaluation. A single factor COX regression analysis was performed to select the genes related to prognosis. A prognostic model was constructed by machine learning, and test whether the model has a test efficacy for prognosis. A chip-in-chip non-small cell lung cancer chemotherapy (NSCLC-ACT) sample was used as a validation dataset for the same validation and prognostic analysis of the model. The coexpression genes of hub genes were obtained by pearson analysis and gene enrichment, function enrichment and protein interaction analysis. The tumor samples of patients with different clinical stages were detected by immunohistochemistry and the expression difference of prognostic genes in tumor tissues of patients with different stages was compared.

**Results:**

By screening, we found that LYN, C3, COPG2IT1, HLA.DQA1, and TNFRSF17 is closely related to prognosis. After machine learning, we constructed the immune prognosis model from these 5 genes, and the model AUC values were greater than 0.9 at three time periods of 1, 3, and 5 years; the total survival period of the low-risk group was significantly better than that of the high-risk group. The results of prognosis analysis in ACT samples were consistent with OBS groups. The coexpression genes are mainly involved B cell receptor signaling pathway and are mainly enriched in apoptotic cell clearance. Prognostic key genes are highly correlated with PDCD1, PDCD1LG2, LAG3, and CTLA4 immune checkpoints. The immunohistochemical results showed that the expression of COPG2IT1 and HLA.DQA1 in stage III increased significantly and the expression of LYN, C3, and TNFRSF17 in stage III decreased significantly compared with that of stage I. The experimental results are consistent with the previous analysis.

**Conclusion:**

LYN, C3, COPG2IT1, LA.DQA1, and NFRSF17 may be new immune markers to judge the prognosis of patients with non-small cell lung cancer.

## Introduction

Non-small cell lung cancer is the most common type of lung cancer, accounting for about 80–85% of the total lung cancer (1). According to statistics from the American Cancer Society, lung cancer accounted for 26% of all cancer deaths in women and 29% of all male deaths in the United States in 2012 (2), and lung cancer is still the number one killer of human cancer-related deaths. In the past few decades, with various surgical methods including surgical resection, chemotherapy, radiation therapy and molecular targeted therapy, the survival rate of patients diagnosed with NSCLC has significantly improved (3). The emergence of gene-targeted therapies such as: EGFR(epidermal growth factor receptor) gene changes and EML4-ALK(spinous cortex microtubule-associated protein-like 4 and anaplastic lymphoma kinase) gene rearrangements has promoted the development of TKI targeted therapy. And tyrosine kinase inhibitors and crizotinib have come to be the important targeted therapies (4–7). But the current effect is still unsatisfactory, and the 5-year overall survival rate of lung cancer is still less than 15% (8, 9). In recent years, immunotherapy has been developed and increasingly used for patients with lung cancer. For example: PD-L1 is overexpressed in many tumor cells including lung cancer cells,and it plays an important role in regulating the immune response of tumor cells (10–12). Currently, there are several clinical trials involving FDA-approved immune checkpoint inhibitors. These trials attack by blocking PD-L1/PD-1 signaling pathway tumor cells expressing PD-L1. However, the current research on prognostic genes related to immune checkpoints is very scarce. Therefore, finding immune prognostic markers in NSCLC is of great significance for the prediction and targeted treatment of NSCLC.

## Materials and Methods

### Data Acquisition and Immune Score

The gene expression profile data set GSE14814 was obtained from the National Biotechnology Information Center (NCBI) GEO (Gene Expression Omnibus) database, which contained 62 samples of untreated cancer patients with non-small cell lung cancer and 62 patients with non-small cell lung cancer after chemotherapy. Samples of 62 untreated patients with non-small cell lung cancer were evaluated for tumor purity scores and immune scores using the Estimate software, and the distribution of immune score was plotted using the ggstatsplot software package in R language. The NSCLC-OBS patients were divided into high and low groups based on the immune scores, using the R language limma package to compare the two groups to obtain differentially expressed genes. “Ggplot2” “heatmap” package in the R software package was used to draw the volcano map and heat map of the difference gene expression.

### Screening of Prognostic Related Genes and GSEA Analysis

For the clinical information of patients with differential genes combined, the unidimensional COX regression analysis was used to reduce the dimension for the first time, and then the second dimension reduction was carried out by 1000 minimum depth method in random forest to obtain the marker with the highest prognostic value gene. Random forest is a method of machine learning, and its purpose is to obtain prognostic value. The largest variable has more accurate results than the conventional multi-factor COX hazard ratio model. The model kernel is a non-linear model, which is closer to the biological reality than the linear model kernel of multi-factor COX regression. In order to obtain a wider range of random forest explanatory variables, and due to the inaccurate biological fitting of single-factor COX regression, the first dimension reduction result was selected, with a *P* value of 0.1, and the obtained variables were included in the second dimension reduction analysis. In order to obtain a more stable learning result, the tree of the random forest was set to 1,000. The result of the learning data also outputs the risk score value of each patient for the next step to show the effect of risk stratification.

Then, GSEA analysis of prognostic marker was performed, and GO and KEGG data sets on the GSEA official website were used for functional enrichment and pathway enrichment. The significant enrichment criteria are that: the absolute value of NES is greater than 1, and the NOMp value is less than 0.05.

### Prediction Model Construction and Evaluation

According to the previous calculation results of COX, GSEA, and random forest model, the immune prognosis model construction and risk assessment were performed for the patients in the OBS group, and the risk scores were ranked. The survminer package in R language was used to find out the best cutting point, and the patients were divided into high and low risk groups for subsequent evaluation of the model efficiency. The expression of five key prognostic genes in the high-risk group and the low-risk group was analyzed and compared, and a heat map was drawn. Based on the results of the high and low risk grouping, the prognostic difference of the K-M survival curve by comparing high and low risk group was plotted, and then the ROC analysis was performed to verify the above results.

### Verification of Prediction Models

The constructed prediction model was applied to a sample of NSCLC-ACT patients, and the score calculations were performed on ACT patients. The expression of five key prognostic genes in the high-risk group and the low-risk group was analyzed and compared, and a heat map was drawn. K-M survival analysis and ROC calculation model accuracy were performed respectively.

### Invasion of Hubgene in Immune Cells and Immune Targets

The GSVA package was used to score the relative infiltration of 24 immune cells in NSCLC-ACT patients, and the NSCLC-ACT patients were divided into high and low risk groups according to the risk grouping results of the random forest model. The infiltration of 24 immune cells in high and low risk groups was compared. Hubgene co-expressed genes were also calculated based on pearson analysis (screening method *P* < 0.05, sorted by R value from small to large). Then, we used R software (3.6.1) cluster analysis package cluster profiler to perform functional enrichment and pathway enrichment analysis on hubgene and its co-expressed genes. After that, we analyzed and calculated the significance of enrichment of 5 prognostic genes and their co-expressed genes in each signal pathway through a hypergeometric distribution exact test, and assessed the signal pathways that were significantly affected (*P* < 0.05). The protein database and the cytoscape software were used to analyze the protein interactions of the hub gene co-expressed genes. In order to further evaluate the relationship between immune factors at the non-cellular level and hubgenes, the correlation analysis between hubgenes and immune checkpoints in the model was performed, and immune targets that were highly correlated with hubgene were screened according to the pearson correlation coefficient.

### Experimental Verification of Prognostic Genes

100 patients diagnosed with stage I and stage III non-small cell lung cancer were selected from our hospital and were divided into two groups. The selected patients had underwent surgery. The general conditions of the two groups of patients are shown in **Table 1**. All patients were diagnosed by pathological examination, and were diagnosed by two senior pathologists. Tissue chips were taken from their tumor tissues. Those tissue chips were first dewaxed for 20 min in xylene and then in fresh xylene. This step was repeat for 1 time. Then, we soaked the dewaxed chip in 100% ethanol for 5 min twice, and then 95% ethanol, 80% ethanol, and distilled water for 5min. Basic antigen repair solution (Tris-E DTA, pH = 9) was used. Then, we put the chips into a pressure cooker to boil them for 2 min, and naturally cool them to room temperature. Then, the chips were incubated for 10 min at room temperature in the dark with 3% H2O2, blocked with normal sheep serum working solution for 30 minutes at room temperature. Then, we added primary antibody RP215, at 4°C overnight. In the next morning, we added HRP-labeled secondary antibody at room temperature for 30 min. DAB coloration and hematoxylin counterstaining was carried. After that, we observed the chips with microscope. We randomly selected 5 high-power fields, and two pathologists independently read the film. The cytoplasmic staining score used four intensity levels: 0: negative; 1, weak positive; 2, moderate positive; 3, strong positive. And four percentage of positive cells: 0, 0%; 1, 1–5%; 2, 6–25%; 3, 26–50%; 4, 51–100%. The final score was the product of intensity grade and positive cell rate (percentage) grade, 0-3 for low expression and 4–9 for high expression.

**Table 1:** Basic information of patients in the stage I lung cancer group and stage III lung cancer group.

Group	Gender		Age	Cancer type	Stage	TNM	Radiotherapy or chemotherapy
	Male	Female					
Stage I lung cancer group	26	24	52.8. ± 1.7	22 cases of LUSC and 28 cases of LUAD	I	T1N0M0	None
Stage III lung cancer group	27	23	54.6 ± 3.2	24 cases of LUSC and 26 cases of LUAD	IIIA	T1N2M0	None
*P* value	>0.05		>0.05	>0.05	/		

### Statistical Analysis

Data analysis was performed using SPSS 20.0 statistical software. Comparison of gene expression levels in different tissues was performed using the χ2 test and independent sample t test, and clinical pathological characteristics were analyzed using the χ2 test. Survival analysis was performed using Kaplan-Meier method. *P <*0.05 was defined as a statistically significant difference.

## Results

### NSCLC-OBS Sample Grouping and Differential Gene Screening

**Figure 1:**
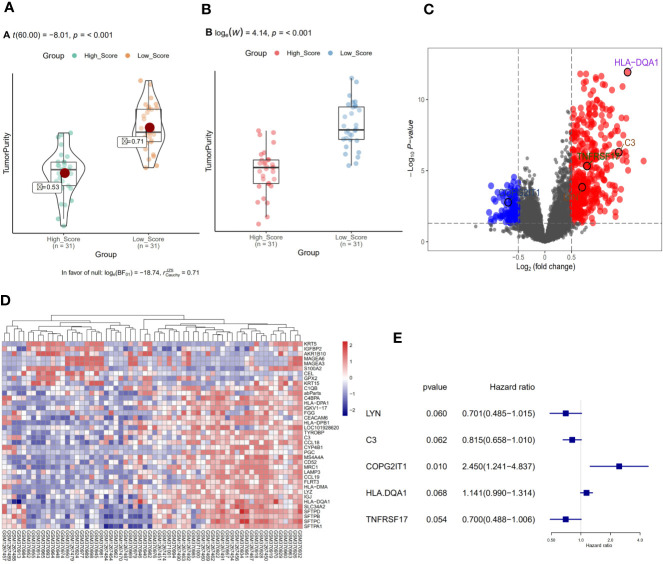
**(A)** Violin distribution of immune scores for OBS samples. **(B)** Comparison of tumor heterogeneity between high and low score groups. **(C)** Volcano map of differential genes in OBS samples. **(D)** Heat map of differential genes in OBS samples. **(E)** Single factor regression analysis of differential genes to screen for key genes for prognosis.

We divided 62 NSCLC-OBS samples into high and low score groups according to their tumor purity and immune scores using the estimation software and the “ggstatsplot” package in R. The violin distribution map of the immune scores (**Figure 1A**), and the T-test results showed the high and low score groups. There were significant differences in tumor heterogeneity (p <0.01) (**Figure 1B**). A differential gene analysis was performed on 62 samples of the high and low score groups, and the results showed that there were 38 differential genes. The R software package was used to draw the volcanic map of the difference results (**Figure 1C**) and heatmap (**Figure 1D**).

### Screening and GSEA Analysis of Prognostic Related Genes

**Figure 2:**
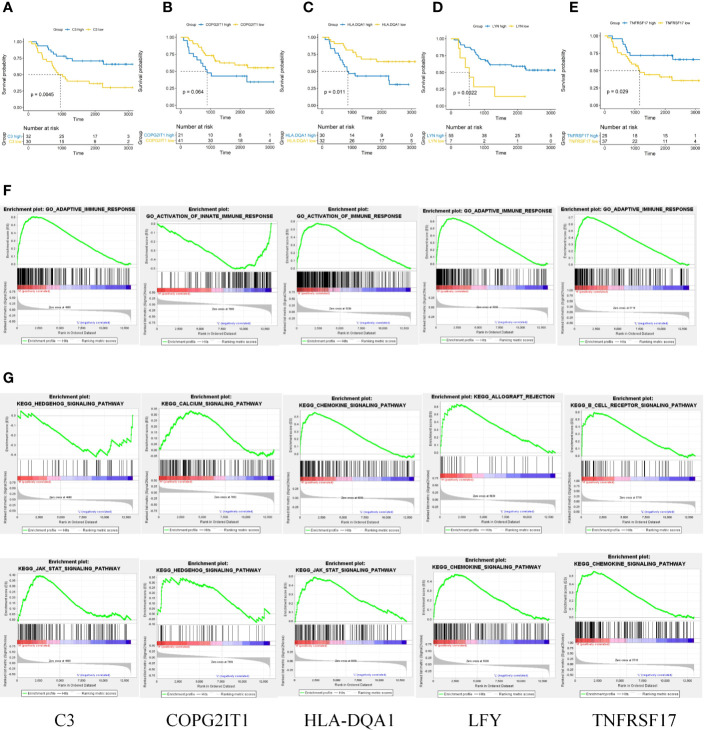
**(A–E)** Prognostic analysis of 5 key prognostic genes. **(F)** GO results of GSEA analysis of five key prognostic genes. **(G)** KEGG results of GSEA analysis of five key prognostic genes.

A single factor COX regression analysis was performed on the differential genes based on the patient’s clinical information, and a total of 95 variables were obtained. After including them in the secondary dimension reduction analysis, we obtained LYN, C3, COPG2IT1, HLA.DQA1, and TNFRSF17 (**Figure 1E**). Expression of these five hubgenes that were most relevant to the prognosis of patients will reduce the survival time of patients (**Figures 2A–E**). The results of GSEA for each prognostic relevant marker were shown in **Figures 2F–G**. As we can see that the GO analysis results of prognostic genes are mainly enriched in ADAPTIVE_IMMUNE_RESPONSE, and the KEGG analysis results are mainly in CHEMOKINE, HEDGEHOG and JAK signaling pathways.

### Construction and Evaluation of Prediction Models

**Figure 3:**
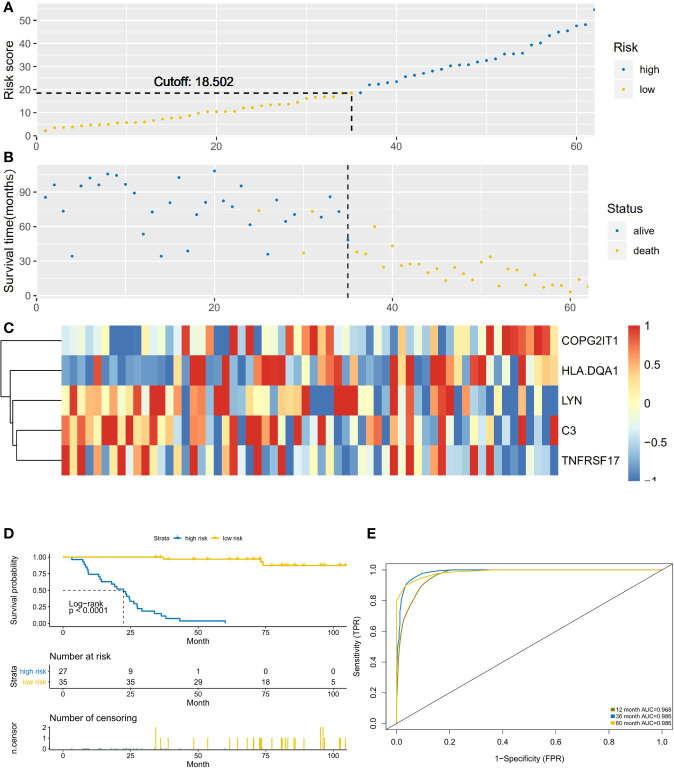
**(A)** The abscissa is the number of patients in OBS group, and the high and low risk groups are divided by the risk score. **(B)** The abscissa is the number of patients, and the division of high-score and low-risk groups is verified by survival. **(C)** Heatmap of the expression of five key prognostic genes in high-risk and low-risk patients in OBS group. **(D)** Comparison of survival analysis between high-risk and low-risk patients; **(E)** ROC analysis test results of model sensitivity and specificity.

According to the previous calculation results, we identified LYN, C3, COPG2IT1, HLA.DQA1, and TNFRSF17 as genes that affected immune prognosis. According to the difference in the expression of these five genes, we divided 62 NSCLC-OBS samples into high and low risk groups (**Figures 3A, B**). Compared with the low-risk group, the gene expression of COPG2IT1 and HLA.DQA1 increased and the gene expression of LYN, C3, and TNFRSF17 decreased in the high-risk group (**Figure 3C**). The K-M survival curve results showed that the survival time and survival rate of patients identified as high-risk group were significantly lower than those of low-risk group (**Figure 3D**). Using ROC to calculate the model, we found that at 1, 3, and 5 years, the AUC values of the prediction models for these three time periods over 5 years were all greater than 0.9 (**Figure 3E**), which proved that this prediction model had a high accuracy in the NSCLC-OBS sample.

### Verification of Prediction Models

**Figure 4:**
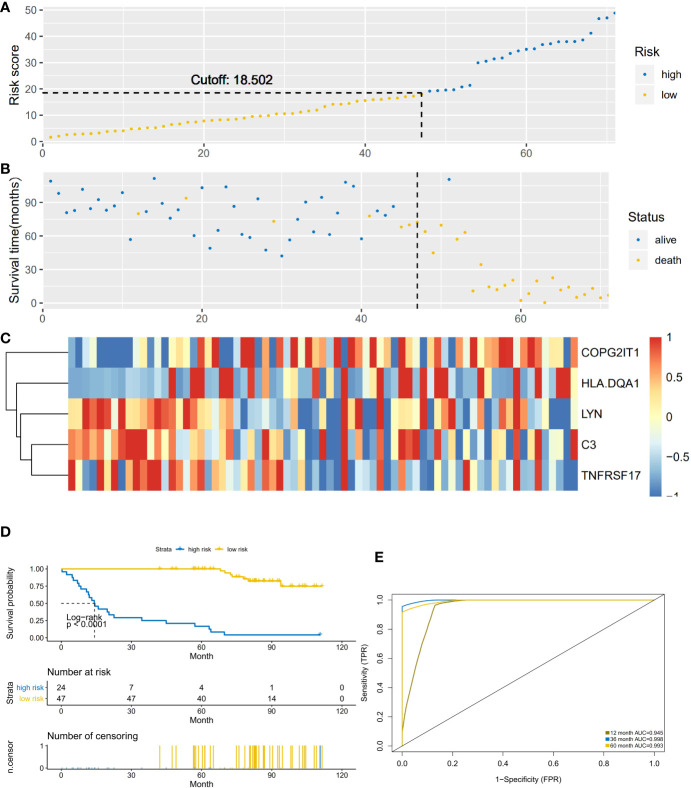
**(A)** The abscissa is the number of patients in ACT group, and the high and low risk groups are divided by the risk score. **(B)** The abscissa is the number of patients, and the division of high-score and low-risk groups is verified by survival. **(C)** Heatmap of the expression of five key prognostic genes in high-risk and low-risk patients in ACT group. **(D)** Comparison of survival analysis between high-risk and low-risk patients. **(E)** ROC analysis test results of model sensitivity and specificity.

This prediction model was applied to 62 NSCLC-ACT samples. The results showed that the high and low-risk groups divided by the model had significant differences (**Figures 4A, B**). Compared with the low-risk group, the gene expression of COPG2IT1 and HLA.DQA1 increased and the gene expression of LYN, C3, and TNFRSF17 decreased in the high-risk group (**Figure 4C**). The survival time and survival rate of patients in the high-risk group were significantly lower than those in the low-risk group. (**Figure 4D**), the ROC curve showed that the AUC value of the prediction model in the three time periods of 1, 3, and 5 years was greater than 0.9 (**Figure 4E**), which proved that this prediction model had high accuracy.

### Model Hubgene Infiltration and Immune Targets in Immune Cells

**Figure 5:**
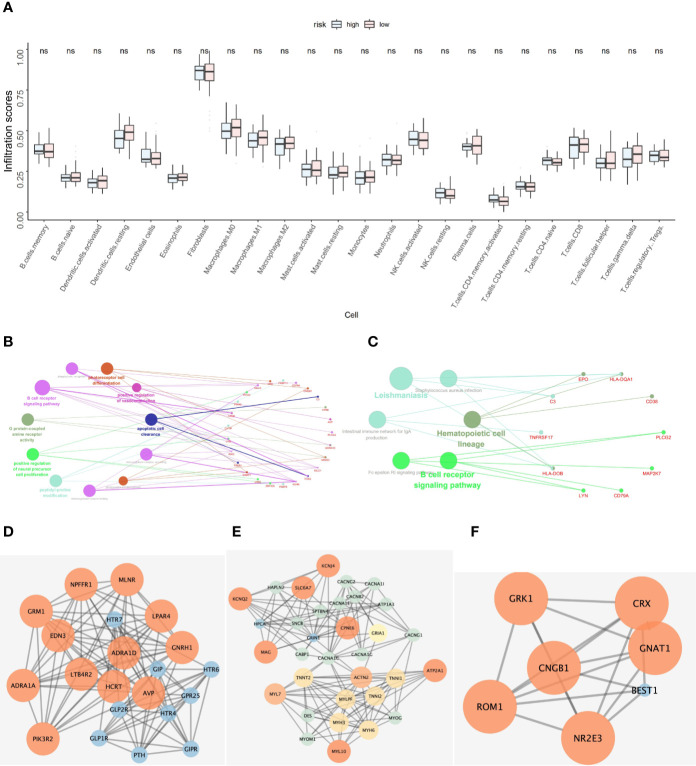
**(A)** Hubgene infiltration in 24 immune cells in high-risk and low-risk groups of ACT patients. **(B)** Functional enrichment results of co-expressed genes of five key prognostic genes. **(C)** Pathway enrichment results of co-expressed genes for five key prognostic genes. **(D–F)** Protein-protein interaction network of co-expressed genes of five key prognostic genes.

The NSCLC-ACT patient samples were evaluated to obtain the relative infiltration scores of 24 immune cells in the NSCLC-ACT patient group. According to the prediction model, the ACT patients were divided into high and low risk groups. After comparing the 24 types of immune cell infiltration in the high and low risk groups, we found that hubgene of the prediction model was most expressed in fibroblasts, but there was no significant difference in immune infiltration between the high and low risk groups in 24 immune cells (**Figure 5A**).

**Figure 6:**
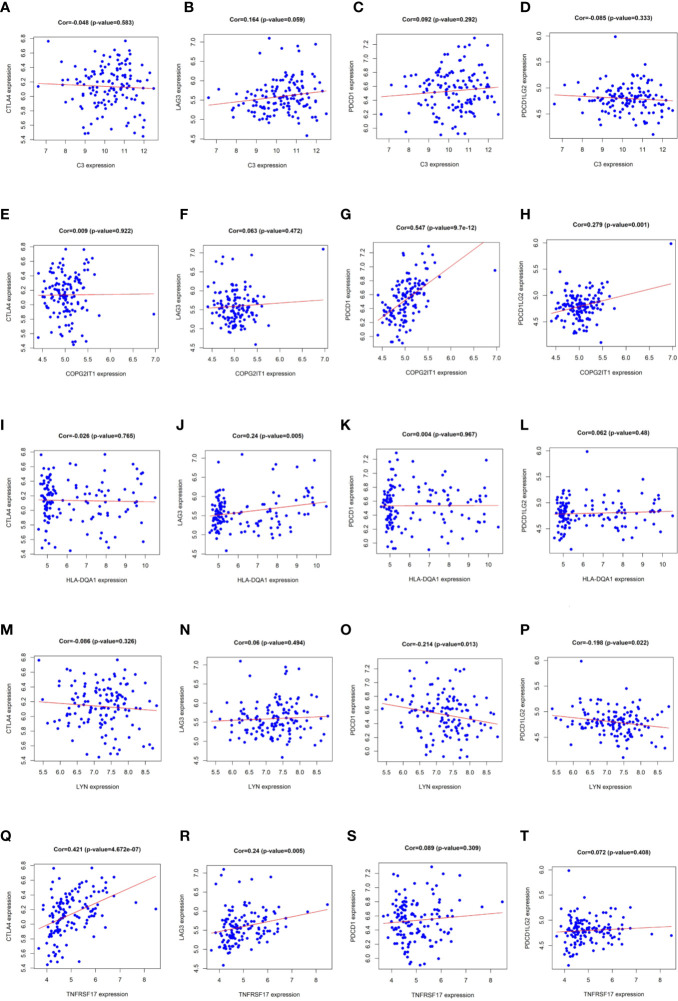
**(A–T) **Correlation analysis results of LYN, C3, COPG2IT1, HLA.DQA1, TNFRSF17 with PDCD1, PDCD1LG2, LAG3 and CTLA4 immune checkpoints.

According to pearson calculation, 100 genes co-expressed with LYN, C3, COPG2IT1, HLA.DQA1, and TNFRSF17 were obtained (p < 0.05, sorted from small to large). Functional enrichment and pathway enrichment analysis of the five hubgenes and 100 co-expressed genes showed that the co-expressed genes were mainly involved in B cell receptor signaling pathway. And these genes were mainly enriched in biological processes such as apoptotic cell clearance, Leishmaniasis, Hematopoietic cell lineage and Intestinal immune network for IgA production (**Figures 5B, C**). Protein interaction analysis of the co-expressed genes of the hub gene yielded three significantly associated protein interaction networks (**Figures 5D–F**). Through the correlation analysis of five hubgene and immune checkpoints in the prediction model, we found that LYN, C3, COPG2IT1, HLA.DQA1, TNFRSF17 were highly correlated with PDCD1, PDCD1LG2, LAG3, CTLA4 immune checkpoints (*p <*0.05) (**Figure 6**).

### Experimental Verification of Prognostic Related Genes

**Figure 7:**
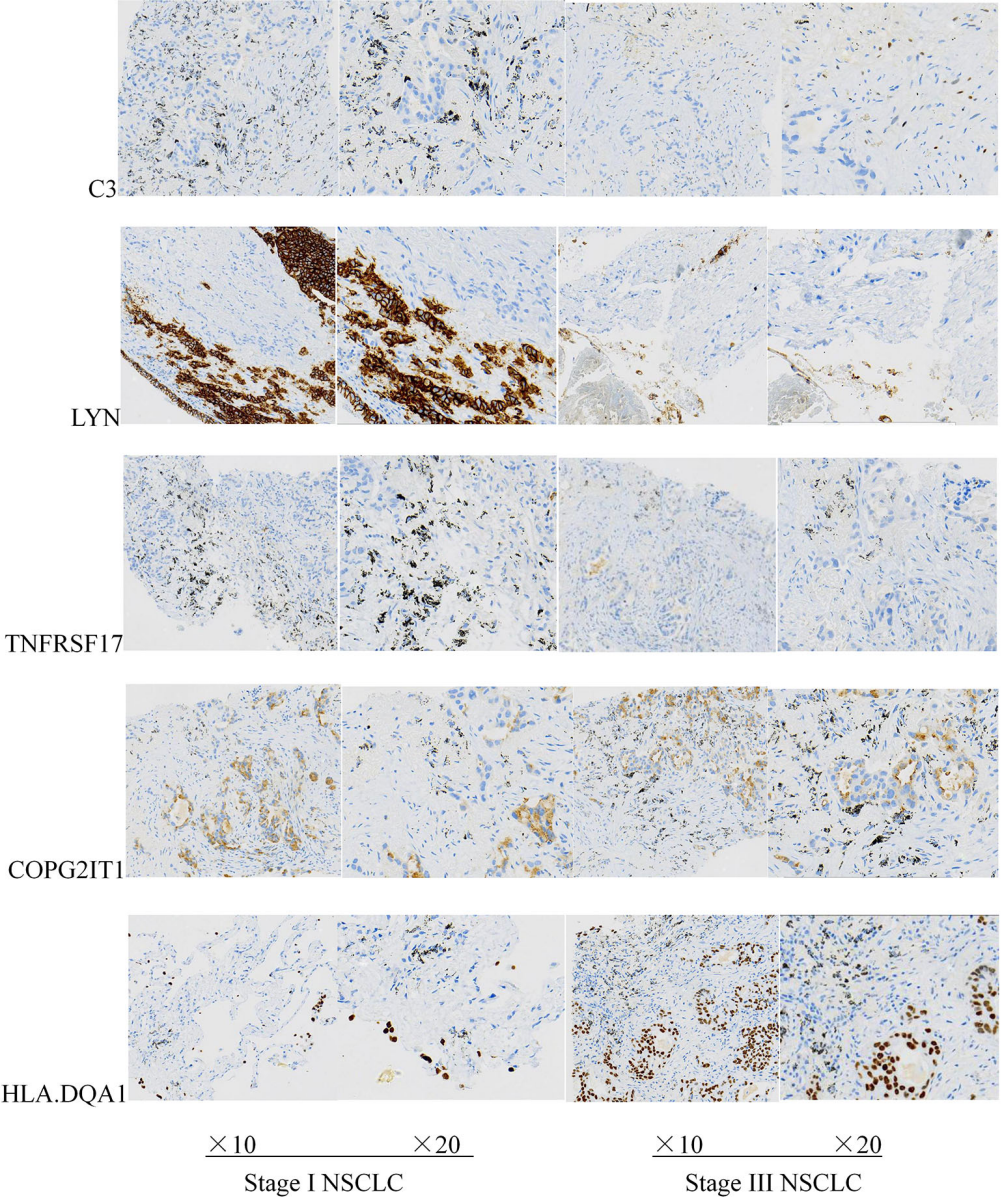
Expression levels of five key prognostic genes in tumor tissues of two groups of patients with NSCLC.

Except for the staging of lung cancer, there was no significant difference in general information between the two groups of patients (*P* < 0.05). The expression levels of five prognostic related genes in the tumor tissues of the two groups of patients are shown in **Table 2**. The results of immunohistochemical analysis showed that compared with the stage I lung cancer group, the expression levels of COPG2IT1 and HLA.DQA1 in the stage III lung cancer group were significantly increased, and the expression levels of LYN, C3 and TNFRSF17 were significantly decreased (**Figure 7**). The results of the immunohistochemical experiments are consistent with the conclusions obtained in the previous analysis.

**Table 2:** Expression of five prognostic related genes in the stage I lung cancer group and stage III lung cancer group.

Gene	Stage I lung cancer group	Stage III lung cancer group	*P* value
LYN	9.3 ± 0.62	2.5 ± 0.36	<0.01
C3	8.9 ± 0.37	2.1 ± 0.35	<0.01
COPG2IT1	3.6 ± 0.59	7.3 ± 0.26	<0.05
HLA.DQA1	4.3 ± 0.87	10.5 ± 1.12	<0.05
TNFRSF17	7.8 ± 0.86	3.2 ± 0.36	<0.05

## Discussion

Non-small cell lung cancer (NSCLC) accounts for about 80–85% of all lung cancers, and the main pathological types include adenocarcinoma and squamous cell carcinoma. For the most part, the treatment of NSCLC depends on the stage at which it is treated. Patient’s surgery resection rate is about 25% (13). However, many patients are still at risk of recurrence of lung cancer after surgical resection. Among patients with NSCLC after surgical resection, the 5-year survival rate of patients in stage I exceeds 70%, but patients in stage IIIA, the 5-year survival rate is only 25% (13–16). Despite the improvement of surgical methods, the combination of radiotherapy and chemotherapy, and the emergence of molecular targeted therapy, the overall 5-year survival rate of lung cancer remains less than 15%. In recent years, emerging immunotherapy performs well in a variety of cancers, including lung cancer. Blocking the PD-L1/PD-1 signaling pathway to attack tumor cells expressing PD-L1 is the current mainstream method. Currently, there are only four types of PD-1/PD-L1 blockers in the clinic, and it is now preferred to use immunotherapies alone or in combination therapy, for example: in one study, TMB was used to predict the effect of PD1 blocker pembrolizumb (keynote-028) on cancer. The results showed that TMB could help to screen patients with more effective anti PD1 treatment (17). Although the anti-cancer activity of PD-1 and PD-L1 inhibitors is exciting, this type of immunotherapy is not effective for all patients, and meta-analyses indicate that a higher risk of rash, thyroid dysfunction, pruritus, pneumonia, and colitis in patients treated with PD-1/PD-L1 inhibitors (18–20). Large amounts of long-term use of PD-1/PD-L1 inhibitors can easily lead to adverse effects such as the expansion of the MDM2 family or distortion of EGFR and resistance to PD-1/PD-L1 inhibitors (21). At present, more and more studies have reported on the prognostic genes of tumors. For example, blocking siglec-15 can amplify the anti-tumor immunity in TME and inhibit tumor growth in some mouse models (22). According to Monkman et al., after high-plex and high-throughput digital spatial Profiling analysis, EpCAM, cytokeratin, ICOS were significantly associated with survival in patients with non-small cell lung cancer (23). However, these are preliminary studies, and for cancer diseases, multi-gene analysis seems to be more effective. Therefore, predicting and finding more biomarkers that may be related to immune prognosis is important for non-small cell lung cancer significance.

We performed immune assessment on untreated NSCLC patient samples and grouped them for differential gene analysis, single-factor COX regression analysis, Receiver Operating Characteristic (ROC) analysis and survival analysis. We identified LYN, C3, COPG2IT1, HLA.DQA1, and TNFRSF17, these five genes may be important immune prognostic genes in NSCLC. We modeled these five genes and applied this model to chemotherapy-treated NSCLC samples, and the results showed that the survival and survival of patients identified as high risk group were significantly lower than those of low-risk group, and ROC analysis of the model shows that AUC values are greater than 0.9, which proves that the model has great credibility. Some literatures show that LYN in allergic airway inflammation can reduce inflammatory cell infiltration and levels of IL-13 and IL-4 and downregulate allergen-induced airway inflammation (24). And C3, HLA.DQA1 has been reported in many reports with glomerular nephropathy, idiopathic membranous nephropathy, steroid-sensitive nephrotic syndrome in children, and other immune system diseases (25–28). It is worth noting that B-cell maturation antigen (BCMA) is a transmembrane glycoprotein in TNFRSF17, and current targeted immunotherapy for BCMA has been used in clinical trials of multiple myeloma with satisfactory results (29–31). Immune checkpoint molecules (immune checkpoint) is a regulatory molecule in the immune system that plays a suppressive role in the immune system and are essential for maintaining autotolerance, preventing autoimmune reactions and minimize tissue damage by controlling the time and intensity of the immune response. The expression of immune checkpoint molecules on immune cells will inhibit the function of immune cells and prevent the body from producing effective anti-tumor immune response, tumor formation immune escape. PDCD1, PDCD1LG2 are the key targets in the treatment of widely used PD-1/PD-L1 inhibitors and plays an important role in adolescent idiopathic arthritis, diffuse large B-cell lymphoma, head and neck squamous cell carcinoma, colorectal cancer and other diseases (32–36). LAG3 is the third clinically targeted inhibitory receptor pathway, it can increase the expression of CD4 +, CD25- and promote T cell dysfunction in tumor microenvironment (37, 38), and is significantly expressed in immunohistochemistry of solid tumors including pancreatic cancer, gastric cancer, colorectal cancer, melanoma, urogenital tract cancer, etc (39). It is worth noting that CTLA4, LAG3, and PD1 seem to interact (40, 41). The combination of anti-PD-1-CTLA4 in the treatment of prostate cancer is gratifying (42); meanwhile, antagonizing LAG3 and PD1 can enhance tumor-specific cellular response and induce tumor rejection (43, 44). However, some studies have shown that the expression of LAG3 in TME of NSCLC samples is decreased (23, 45). Whether this indicates that LAG3 plays a unique role in NSCLC, but its specific mechanism and target are still unknown. Correlation analysis of the five hubgene genes and immune checkpoints, LYN, C3, COPG2IT1, HLA.DQA1, and TNFRSF17 highly correlated with PDCD1, PDCD1LG2, LAG3, and CTLA4 immune checkpoints. This indicates that these five hubgene genes may affect the disease progression of NSCLC patients by regulating immune checkpoints expression. Co-expression analysis of these five hubgene genes yields 100 co-expressed genes (p < 0.05). Performing functional enrichment and pathway enrichment analysis of hubgene and its co-expressed genes, we found that the GO prognostic gene were mainly enriched in ADAPTIVE_IMMUNE_RESPONSE, and KEGG was mainly enriched in CHEMOKINE, HEDGEHOG, and JAK signal pathways. Co-expressed genes are mainly involved in the B cell receptor signaling pathway, and are mainly enriched in apoptotic cell clearance, leishmaniasis hematopoietic cell lineage, intestinal immune network for IgA production, and other biological processes. Among them, CHEMOKINE is an emerging family of chemokine cytokines, which shows a wide range of functions, such as regulating steady-state leukocytes flow and development, and activating the innate immune system. Inappropriate balance of chemokine synthesis or chemokine receptor expression can lead to a variety of pathological diseases. Some drugs containing chemokine-derived peptides may exert antitumor activity in lung cancer, prostate cancer, colon cancer, melanoma, and breast cancer by affecting the tumor microenvironment (46). And adaptive immune response B cell receptor signaling pathway and adaptive cell clearance all play important roles in the process of tumorigenesis and development.

The B cell receptor (BCR) pathway has been identified as a potential therapeutic target in a number of common B cell malignancies. We found that screened five prognosis-related genes were all closely related to immunity response and mainly involved in B cell receptor signaling pathway. Tyrosine protein kinase Lyn is a protein encoded by the LYN gene in humans and a member of the Src protein tyrosine kinase family. Among various hematopoietic cells, Lyn has become a key enzyme involved in the regulation of cell activation. In these cells, a small amount of LYN is associated with cell surface receptor proteins, including B cell antigen receptor (BCR), CD40 or CD19. Yang et al. (47) found that SHP-1 deficiency in B-lineage cells was associated with heightened lyn protein expression and increased lyn kinase activity. In their study, a modest increase in p56/53lyn protein expression was detected in primary spleen B cells of motheaten mice. Their study suggested that SHP-1 deficiency in B-lineage cells, especially pre-B cells, played an important role in regulating lyn through a post-transcriptional mechanism.

Yamamoto et al. (48) found that the Src-family protein tyrosine kinase Lyn (p56lyn and p53lyn) was expressed preferentially in B cells and Lyn was likely to participate in B-cell antigen receptor-mediated signaling. Yamanashi et al. (49) reported that the Src-like protein-tyrosine kinase p56/p53lyn associates with cell membranes and transduces signals from activated cell surface receptors. p56lyn and p53lyn, products of alternatively spliced lyn mRNA, had differential responses on stimulation of B-cell antigen receptor. Werner et al. (50) firstly described a B cell population containing high levels of intracellular C3, suggesting a new role of B cells in the maintenance of the inflammation by complement C3. Kulik et al. (51) found that when CR2 was bound by its primary C3 activation fragment-derived ligand, designated C3d, it coassociated with CD19 on B cells to amplify BCR signaling. Spaapen et al. (52) firstly reported the hematopoietic mHag presented by HLA class II (HLA-DQA1*05/B1*02) molecules to CD4(+) T cells. This antigen was encoded by a single-nucleotide polymorphism (SNP) in the B cell lineage-specific CD19 gene, which was an important target antigen for immunotherapy of most B cell malignancies.

Mutations in the HLA class II genes could lead to loss of expression of HLA-DR and HLA-DQ in diffuse large B-cell lymphoma (53). In current study, differential expression of HLA-DQA1 gene alleles was analyzed in three different cell populations isolated from peripheral blood B lymphocytes, monocytes, and whole-blood cells. DQA1*03 alleles were among the most expressed in all cell types, whereas DQA1*05 alleles were least expressed in whole blood and monocytes and among the most expressed in B cells (54). It was reported (55) that TNFSF13 could support leukemia cell proliferation in an NF-κB-dependent manner by binding TNFRSF17 and suppressed apoptosis. Kampa et al. (56) had already overviewed the whole tumor necrosis factor system and focused on A proliferation-inducing ligand (APRIL, TNFSF13) and B cell-activating factor (BAFF, TNFSF13B) and their receptors transmembrane activator and Ca modulator (CAML) interactor (TACI, TNFRSF13B), B cell maturation antigen (BCMA, TNFRSF17), and BAFF receptor (BAFFR, TNFRSF13C).

## Conclusion

In conclusion, our research indicates that LYN, C3, COPG2ITL, HLA.DQAL, and TNFRSFL17 are potential prognostic markers for non-small cell lung cancer. The results of immunohistochemical experiments of patients’ pathological tissues are consistent with this conclusion. These prognosis-related genes are mainly enriched in B cell receptor signaling pathways and are highly related to PDCD1, PDCD1LG2, LAG3, and CTLA4 immune checkpoints; this suggests that immunotherapy may improve the prognosis of non-small cell lung cancer patients by regulating these prognosis-related genes.

## Data Availability Statement

Publicly available datasets were analyzed in this study. This data can be found here: the Gene Expression Omnibus (GSE14814).

## Ethics Statement

The studies involving human participants were reviewed and approved by The Ethics Committee of the First Affiliated Hospital of Nanchang University. The patients/participants provided their written informed consent to participate in this study.

## Author Contributions

JX research the design and drafted the manuscript. HN experiment implementation. JH: help modify articles. XW: literature search. KL experiment implementation. LT help modify articles and collate references. ZX review and revision of the manuscript and writing guidance. All authors contributed to the article and approved the submitted version.

## Conflict of Interest

The authors declare that the research was conducted in the absence of any commercial or financial relationships that could be construed as a potential conflict of interest.
